# Climate-driven shifts in avocado suitability zones in India: Insights from ensemble modelling and niche hypervolume

**DOI:** 10.1371/journal.pone.0338518

**Published:** 2026-01-14

**Authors:** Karunakaran G., Manish Mathur, Kanupriya C., Senthilkumar M., Sakthivel T., Kadirvel G., Murlidhara B.M., Kavino M., Hazarika T.K., Ruchitha T.

**Affiliations:** 1 Division of Fruit Crops, ICAR-Indian Institute of Horticultural Research, Bengaluru, Karnataka, India; 2 ICAR Central Arid Zone Research Institute, Jodhpur, Rajasthan, India; 3 Central Coffee Research Institute, Coffee Research Station Post, Chikkamagaluru, Karnataka, India; 4 ICAR-Agricultural Technology Application Research Institute, Zone VI, Guwahati, Assam, India; 5 ICAR-Indian Institute of Horticultural Research, Central Horticultural Experimental Station, Chettalli, Kodagu, Karnataka, India; 6 Department of Fruit Science, Horticultural College and Research Institute, Tamil Nadu Agricultural University, Coimbatore, TamilNadu, India; 7 Department of Horticulture Aromatic and Medicinal Plants, Mizoram University, Aizawl, Mizoram, India; Canakkale Onsekiz Mart University, TÜRKIYE

## Abstract

Avocado (*Persea americana* Mill.), a nutrient-rich tropical fruit, is gaining prominence in India due to rising domestic demand and export potential. However, its cultivation remains fragmented, largely confined to southern states, with limited knowledge of ecological requirements under diverse agro-climatic zones and climate change scenarios. This study aimed to identify key bioclimatic and non-bioclimatic factors influencing avocado suitability, model its current and future distribution using ensemble species distribution modelling (ESDM), assess niche dynamics under four Representative Concentration Pathways (RCPs 2.6, 4.5, 6.0, and 8.5) for 2050 and 2070, and evaluate implications for climate-resilient agroforestry planning. Using 35 spatially thinned occurrence records and high-resolution environmental predictors, ESDM integrating eight machine learning algorithms was applied. Model performance was robust (AUC: 0.86–0.91), with Random Forest and Maxent performing best. Critical predictors included isothermality, minimum temperature of the coldest month, precipitation in the coldest quarter, urbanization, and forest cover. Current suitability hotspots were concentrated in Kerala and Tamil Nadu. Future projections under RCPs 2.6 and 6.0 indicated northward and altitudinal expansion into the Western Ghats, northeastern hills, and eastern India, whereas RCP 8.5 suggested increased fragmentation and instability. Niche analysis revealed ecological breadth expansion under low to moderate emissions, but contraction and displacement under high-emission conditions. These findings highlight scope for expanding avocado cultivation under low to moderate emissions, provided thermal and precipitation stability is maintained. The study offers a geospatial foundation for climate-smart avocado production, conservation, and policy, emphasizing the protection of climatic refugia in southern India and adaptive agroecological strategies for long-term sustainability.

## Introduction

Avocado (*Persea americana* Mill.), a member of the Lauraceae family, is an economically and nutritionally significant fruit crop. Indigenous to the tropical highlands of Central and South America, with southern Mexico and Guatemala as primary centres of genetic variation [1], the species comprises three horticultural varieties—Mexican (*P. americana* var. *drymifolia*), Guatemalan (*P. americana* var. *guatemalensis*), and West Indian (*P. americana* var. *americana*)—each adapted to distinct morphological and ecological conditions [2–4]. Hybrid cultivars, especially ‘Hass’, dominate the global market owing to superior postharvest longevity and consumer preference [5].

Global avocado production has exceeded 8.97 million metric tons in recent decades [6]. Rising demand reflects its recognition as a “superfood,” valued for monounsaturated fats, dietary fibre, and essential vitamins (E, K, B6) [7,8]. The global avocado market, valued at USD 14.9 billion in 2023, is projected to expand at a CAGR of 5.8% from 2024–2032, with strong growth anticipated in Asian and Middle Eastern markets [9].

In India, avocado was introduced in the early 20th century in Karnataka, Tamil Nadu, and Kerala [10,11]. For decades, cultivation remained limited to homestead gardens or intercropping with coffee, but increasing health awareness and demand for crop diversification have renewed interest in the crop [12]. Domestic production, ~ 7,000 tons annually, is concentrated in Karnataka, Tamil Nadu, and parts of the Northeast [13]. Despite promising grafted cultivars such as ‘Arka Supreme’ and ‘Arka Coorg Ravi’ developed by ICAR-IIHR [14], production remains fragmented and heavily reliant on seed propagation. Imports, estimated at 1,200 tonnes annually from Kenya and New Zealand [15], supply major urban markets where retail prices for imported *Hass* range from ₹200–500/kg [16], underscoring the gap between demand and domestic availability.

Ecological mismatches increasingly constrain avocado expansion in India. The species has a narrow precipitation optimum and is sensitive to low temperatures and irregular rainfall; both excess and deficit precipitation can reduce yield and fruit quality [17]. By 2050, global prime avocado-growing regions are projected to decline by 14–41% under climate change scenarios. In India, while quantitative yield-loss estimates remain limited, high humidity and rainfall often favor anthracnose, reducing yields by up to ~30%. Orchards in non-optimal zones exposed to rainfall or temperature extremes may therefore face yield penalties of 20–30% or more compared with orchards in optimal zones. Optimal conditions typically include subtropical to temperate climates with 1,000–2,500 mm/year rainfall, well-drained soils, and altitudes of 1,400–2,500 m [18]. Orchards outside these thresholds often show lower yields, poor fruit quality, and higher pest and disease incidence [19–21]. Additional barriers include reliance on seed propagation, lack of standardized planting material [12], and weak postharvest infrastructure, with cold-chain and ripening constraints causing up to 30% losses [16].

Similar challenges have been observed in other tropical regions. In Colombia, unregulated *Hass* expansion into high-rainfall Pacific regions led to major crop failures from moisture stress [22]. To address such risks, Ecological Niche Modelling (ENM) has been widely applied for identifying suitable cultivation zones. ENM predicts potential habitats from occurrence records and environmental variables such as temperature, precipitation, and soil properties [23,24]. In Colombia, ENM-guided zoning of Andean regions (1,400–2,500 masl) improved avocado yields by 20–30% while avoiding unsuitable lowlands [20]. In Kenya, ENM identified optimal zones in the Rift Valley, facilitating export growth [25]. These examples highlight ENM’s utility for sustainable avocado production.

In India, however, ENM applications for avocado remain limited. The recent work [26] represents an important early effort, applying a single-algorithm climatic niche model (MaxEnt) to predict potential distribution in southern India. While informative, that study focused primarily on climatic envelopes and did not integrate non-bioclimatic factors such as soil, topography, or land-use pressures, nor did it evaluate multiple climate change scenarios. In contrast, the present study advances this framework by employing an ensemble species distribution modelling (ESDM) approach that integrates eight machine learning algorithms and incorporates both bioclimatic and non-bioclimatic variables, thereby enhancing robustness and ecological realism. Moreover, by coupling ESDM with ellipsoidal niche hypervolume analysis, we quantify niche breadth, centroid shifts, and ecological expansion/contraction across four Representative Concentration Pathways (RCPs), providing the first such integrated assessment for avocado in India. This methodological synthesis bridges the spatial and analytical gaps of earlier research and establishes a reproducible framework for climate-resilient avocado planning at a national scale.

Given India’s diverse agro-ecological landscapes—from the humid Western Ghats and Nilgiri Hills to the temperate Himalayan foothills—ENM offers a strategic tool for aligning avocado cultivation with climatic and landscape suitability. Accordingly, this study aims to: (1) identify the bioclimatic, edaphic, and land-use factors most influential for avocado suitability across Indian agro-climatic zones; (2) map potential ecological niches using occurrence records and high-resolution environmental data; (3) analyze existing production regions against projected suitability to identify gaps and underutilized high-potential areas; (4) delineate priority regions for climate-resilient avocado development, focusing on the Himalayan foothills, Western Ghats, and Northeastern hill states; and (5) develop a spatially explicit decision-support framework to guide evidence-based planning for avocado production and reduce import reliance.

To our knowledge, this is the first study to present a spatially explicit ecological niche and hypervolume assessment of avocado in India. By integrating climatic, edaphic, and land-use variables across multiple RCP scenarios, we quantify future suitability shifts, niche expansion, and refugia stability. This approach provides a reproducible and practical framework to inform policymakers, researchers, and farmers in advancing climate-smart avocado cultivation.

## Data and methods

### Species presence records

**Fig 1:**
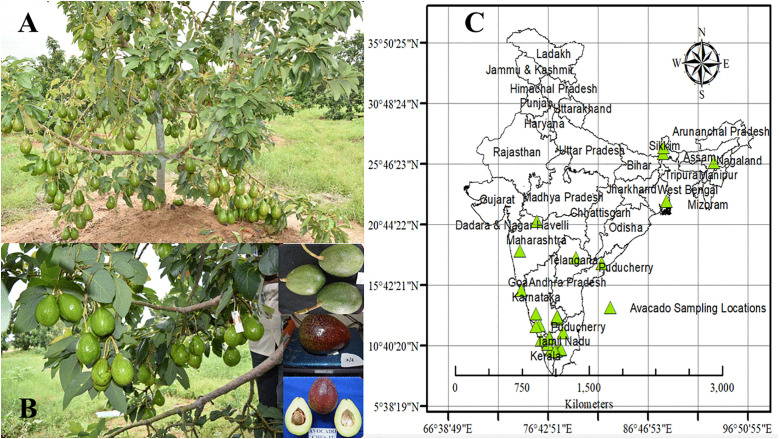
Habit of Avocado plant (A), fruits, the commercial part of this species (B) and sampling location of avocado trees in India (C).

Species occurrence data were compiled from multiple sources to ensure comprehensive coverage of the distribution range. Primary datasets included: (1) the Global Biodiversity Information Facility (GBIF; https://doi.org/10.15468/dl.pqrqqt, accessed 23 April 2025), (2) the GeoCAT platform, which incorporates iNaturalist and Flickr observations [26], accessed 3 May 2025, and (3) field surveys conducted between 2022 and 2024 across five Indian states—Karnataka, Kerala, Tamil Nadu, Andhra Pradesh, and Sikkim (S1 Table, Fig 1). These were further supplemented with occurrence records reported in published literature [27,28]. All georeferenced records were standardized to the WGS84 coordinate system and cross-verified using high-resolution Google Earth satellite imagery to ensure accuracy. Spatial analysis and mapping were performed using ArcMap GIS software (version 10.8) [29]. To minimize redundancy, duplicate records were removed, and spatial thinning was applied with the *spThin* package in R [30]. This procedure reduced spatial autocorrelation, ensuring statistical independence among occurrence points. The curated dataset was stored and analyzed in a structured CSV format to facilitate species distribution modelling (SDM) and ecological niche analysis. After quality control, a total of 35 spatially independent and accurately georeferenced records were retained for modelling. A thinning distance of 25 km^2^ was applied following [31]. Given the small sample size, additional anti-overfitting measures were applied, including regularization tuning in MaxEnt (via ENMeval) and limiting the number of predictors through variance inflation factor (VIF < 10) filtering. This conservative approach prioritized data accuracy and reduction of spatial autocorrelation, which are widely recognized as more critical for reliable SDM performance than maximizing the sheer volume of occurrence records.

## Environmental gradients

### Bioclimatic (Bio) variables

Bioclimatic variables for distribution modelling were obtained from WorldClim version 1.4 (https://worldclim.org/data/cmip6/cmip6clim30s.html). All nineteen standard bioclimatic variables were extracted for both current conditions (1970–2000 average) and future climate projections. Future climate data covered two time periods (2041–2060 [2050 average] and 2061–2080 [2070 average]) under four Representative Concentration Pathways (RCPs 2.6, 4.5, 6.0, and 8.5) [32]. These RCPs represent different radiative forcing scenarios (W/m^2^) by 2100:

RCP 2.6: peak emissions between 2010–2020 followed by decline [33].
RCP 4.5: peak emissions around 2040 followed by decline.
RCP 6.0: transitional scenario with gradual emission reductions.
RCP 8.5: business-as-usual trajectory with continuous emission increase [34].

All climate layers were converted into ASCII format using DIVA-GIS version 7.5 [29] for compatibility with the modelling workflow. A complete list of bioclimatic variables used in the analysis is provided in S1 Table.

### Soil variables (100–200 cm depth)

Soil parameters were obtained from ISRIC’s SoilGrids database (https://isric.org/soilgrids, accessed 15 August 2024). Seven key soil properties were extracted at standardized depths: bulk density (kg cm ⁻ ^3^), cation exchange capacity (cmol kg ⁻ ^1^), soil pH (H₂O), soil texture (% sand, silt, clay), soil organic carbon stock, and nitrogen content (g kg ⁻ ^1^). Soil layers were retrieved via WMS servers and processed using ArcMap GIS software (version 10.8). Data extraction adhered to SoilGrids protocols [35]. To ensure compatibility with ecological niche modelling, soil variables were resampled to the spatial resolution of the bioclimatic datasets (30 arc-seconds). Because avocado is a deep-rooted species, with roots extending beyond 1.5 m, soil properties at depths of 100–200 cm are particularly critical, influencing water retention and nutrient uptake [36]. Incorporating these deeper soil layers enabled the analysis to capture the environmental conditions most relevant to avocado growth and resilience, rather than relying solely on surface soil characteristics.

### Land use and land cover (LULC)

Land use and land cover (LULC) predictors were incorporated to account for habitat and anthropogenic influences. LULC data were sourced from the European Space Agency Climate Change Initiative (ESA CCI-LC, 2016, 300 m resolution), accessed via https://www.esa-landcover-cci.org. Variables included forest, grass/shrub/woodland (GRS), barren or sparsely vegetated areas, urbanized/built-up land (residential and infrastructure), croplands (irrigated and rainfed), and water bodies [37]. The classification followed the UN Land Cover Classification System (LCCS), and rasters were resampled to 30 arc-seconds to match the climatic grid.

### Human modification of terrestrial systems (HMTS)

The Human Modification of Terrestrial Systems (HMTS) dataset, available at a 1 km^2^ resolution, quantifies anthropogenic impact across all continents except Antarctica. It applies a continuous scale (0–1) to measure alterations in terrestrial landscapes, integrating thirteen human-induced stressors through advanced modelling techniques. Spatially explicit datasets from the median year 2016 were used for analysis [38,39]. The HMTS dataset, in GeoTIFF format, was obtained from the Socioeconomic Data and Applications Center (SEDAC). This layer was incorporated to evaluate how anthropogenic pressures influence avocado distribution dynamics.

### Habitat heterogeneity index (HHI)

Habitat heterogeneity reflects the diversity of environmental and geographical characteristics within an area. Traditional assessments, often based on field surveys, are labour-intensive and spatially limited [40]. To overcome this, [41] developed 14 global habitat heterogeneity metrics at 1 km resolution using remotely sensed Enhanced Vegetation Index (EVI) data from MODIS. EVI improves upon NDVI by reducing atmospheric and soil background noise. Texture metrics derived from EVI capture spatial variability in vegetation, including average density (standard deviation), diversity (contrast), unpredictability (entropy), homogeneity (Simpson and evenness), and spatial dependence. In this study, we used EVI-derived indices at ~1 km resolution (30 arc-seconds)—coefficient of variation, evenness, range, Shannon and Simpson diversity, standard deviation, and uniformity—to link *P. americana* habitat suitability with broader vegetation and community dynamics. These standardized indices provide deeper insights into biodiversity patterns and facilitate the identification of conservation-relevant heterogeneity.

### Slope and elevation

Elevation and slope data were derived from the Shuttle Radar Topography Mission (SRTM), providing high-resolution digital elevation models (DEM) at 3 arc-second resolution. These topographic parameters are critical for SDM as they strongly influence microclimate, soil formation, and hydrological processes [42,43]. Slope classes were delineated as follows: C1: 0–0.5%, C2: 0–2%, C3: 2–5%, C4: 5–10%. Together with elevation data, these classes were incorporated to refine assessments of habitat suitability for avocado.

### Issue of multicollinearity

Multicollinearity in species distribution modelling (SDM) occurs when predictor variables are highly correlated, leading to inflated coefficient variance and overfitting [44]. We unified the collinearity criterion using Pearson’s correlation coefficient (r ≥ 0.85) as the threshold for variable exclusion. Highly correlated variables were clustered, and from each cluster, a single variable was retained based on ecological interpretability. The final retained predictors included Bio-3 (Isothermality), Bio-4 (Temperature Seasonality), Bio-6 (Minimum Temperature of the Coldest Month), Bio-13 (Precipitation of the Wettest Month), Bio-16 (Precipitation of the Wettest Quarter), and Bio-19 (Precipitation of the Coldest Quarter) for bioclimatic datasets. Non-bioclimatic variables retained were forest cover, urbanization, rainfed cropland, bulk density, and soil organic carbon.

### Ensemble species distribution modelling

Species distribution modelling (SDM) was implemented in R 4.3.1 using the SSDM, biomod2, dismo, and maxnet packages. The ensemble combined outputs from eight algorithms—Support Vector Machines (SVM), Generalized Linear Models (GLM), Generalized Additive Models (GAM), Multivariate Adaptive Regression Splines (MARS), Classification Tree Analysis (CTA), Artificial Neural Networks (ANN), MaxEnt, and Random Forest (RF)**—each calibrated with tuned parameters. Models were trained using 70% of occurrence data and validated on 30%, with 10-fold cross-validation and five pseudo-absence replicates per algorithm. Ensemble projections were weighted by individual model AUC performance scores, not by simple averaging, to ensure that well-performing models contributed proportionally more to final suitability predictions. Model accuracy was assessed using AUC, sensitivity, specificity, Kappa, and True Skill Statistic (TSS) values, and Variable Importance Percentage (VIP) was computed to identify key environmental predictors [45–52].

### Elimination of uncertainty and habitat suitability

Habitat suitability classes were defined using statistically derived thresholds based on the maximum TSS and equal sensitivity–specificity criteria. The ESDM tool generated two raster outputs: (1) species occurrence probability and (2) associated uncertainty. Both were imported into ArcMap, where the Raster Calculator (Spatial Analyst Tool) excluded ambiguous regions. Suitability categories were defined as: excellent (0.80–1.00), moderate (0.60–0.79), marginal (0.40–0.59), and low (<0.40). The spatial extent (km^2^) of each class was calculated in ArcGIS. Future climate scenarios were then assessed for percentage changes in optimal and intermediate suitability following [53–55].

### Percent of indigenous and ellipsoid niche hypervolume

The Percent Indigeneity (PI) was calculated following [49], with suitability weights assigned as optimum = 1.0, moderate = 0.75, marginal = 0.50, and low = 0.25. A higher PI value indicates broader ecological generality, while a lower value reflects ecological restriction. Ellipsoidal niche hypervolumes were constructed using the NicheToolBox (Ntbox) package in R, employing the three most influential predictors per RCP and variable set. The models were parameterized with the centroid and covariance matrix of the species’ environmental space, enabling visualization of niche expansion and contraction across temporal scenarios.

## Results

A total of 35 georeferenced occurrence records for avocado were compiled from multiple sources (Fig 1). To address multicollinearity, several bioclimatic variables were excluded from the analysis. Specifically, Bio-10, Bio-11, Bio-12, Bio-14, and Bio-18 were removed from both current and future datasets (2050 and 2070); Bio-1 and Bio-2 were excluded from all RCP scenarios (2050 and 2070); and Bio-3, Bio-4, Bio-7, and Bio-8 were eliminated from the current dataset.

### Model performance

The assessment of Ensemble Species Distribution Modelling (ESDM) under present and future climate scenarios (RCP 2.6, 4.5, 6.0, and 8.5 for 2050 and 2070) demonstrated consistently strong predictive efficacy. Model evaluation metrics—including Area Under the Curve (AUC), sensitivity, specificity, Kappa, and True Skill Statistic (TSS)—indicated robust performance across all scenarios (Table 1). Together, these measures reflect the model’s accuracy, its ability to correctly predict presence (sensitivity) and absence (specificity), and the agreement between predicted and observed distributions (Kappa and TSS). Algorithm-specific performance is detailed in S2 to S4 Tables. All eight algorithms exhibited commendable accuracy, with AUC scores ranging from 0.81 to 0.90, and Random Forest consistently producing the highest AUC among predictor sets. The ensemble models yielded AUC values between 0.86 and 0.91 across scenarios. The present climate model achieved an AUC of 0.87, signifying strong discriminatory capacity between suitable and unsuitable habitats. Among the future projections, the 2050 RCP 2.6 scenario achieved the highest climate-based AUC (0.90), reflecting slightly improved accuracy under a low-emission trajectory, likely due to clearer habitat suitability signals under mild climate change. The highest overall AUC (0.91) was obtained with the non-bioclimatic (NBC) predictor set. Uncertainty maps associated with each predictor set are presented in S1 Fig.

**Table 1:** Algorithm evaluation parameters of Ensemble Species Distribution Modelling.

Environmental Gradients	AUC	Sensitivity	Specificity	Kappa	TSS
Current	0.87	0.85	0.82	0.48	0.67
2050 RCP 2.6	0.90	0.87	0.89	0.58	0.75
2050 RCP 4.5	0.88	0.84	0.88	0.58	0.72
2050 RCP 6.0	0.89	0.86	0.87	0.56	0.73
2050 RCP 8.5	0.88	0.85	0.87	0.57	0.71
2070 RCP 2.6	0.86	0.84	0.84	0.53	0.68
2070 RCP 4.5	0.88	0.84	0.85	0.52	0.69
2070 RCP 6.0	0.88	0.83	0.90	0.59	0.73
2070 RCP 8.5	0.87	0.84	0.86	0.55	0.71
NBC	0.91	0.90	0.90	0.60	0.80

### Variable importance per centage

The analysis of variable importance across present and future bioclimatic scenarios revealed notable shifts in the key environmental determinants influencing avocado distribution, reflecting adaptive responses of suitable habitats to climate change. Under current climatic conditions, the most influential predictors were Bio-19 (precipitation of the coldest quarter), Bio-6 (minimum temperature of the coldest month), and Bio-17 (precipitation of the driest quarter) (Table 2). These findings emphasize the species’ dependence on cold-season precipitation and minimum temperature thresholds for sustaining viable habitats. By contrast, future climate projections (2050 onward) indicated a substantial reordering of predictor importance. Across all RCP scenarios (2.6, 4.5, 6.0, and 8.5), Bio-3 (Isothermality) consistently emerged as the dominant variable, contributing up to 35.86% under the 2050 RCP 8.5 scenario. This highlights the increasing role of temperature uniformity and intra-annual thermal stability in determining habitat suitability under changing climates. Other variables with moderate to high influence across scenarios included Bio-13 (precipitation of the wettest month), Bio-16 (precipitation of the wettest quarter), and Bio-4 (temperature seasonality), suggesting that seasonal climatic extremes will play a greater role in shaping avocado distribution under future climates. A comparative analysis indicates a clear transition in ecological determinants—from reliance on cold- and dry-season conditions in the present to dependence on thermal stability and seasonal precipitation regimes in the future. Temperature extremes, represented by Bio-6, Bio-7, and Bio-4, were particularly influential under mid- to high-emission pathways (RCP 4.5 and RCP 6.0), underscoring the increasing importance of thermal stress in shaping species niches as global warming intensifies. These findings reinforce the need to prioritize microclimatic stability, soil moisture retention, and seasonal buffer zones in conservation planning and agroforestry strategies, particularly under elevated RCP scenarios that push ecosystems toward climatic extremes and modified biological niches. Non-bioclimatic variables also played a significant role in shaping habitat suitability (S2 Fig.). Among these, urbanization (VIP = 22.59), forest cover (VIP = 6.26), and rainfed cultivation (VIP = 5.62) exerted the greatest influence, reflecting the interplay between anthropogenic pressures, land use, and ecological suitability for avocado.

**Table 2:** Variable Importance Values of Bio-climatic variables with different temporal and RCPs scenarios.

Bio-Climatic Variables	Current	2050 RCPs				2070 RCPs			
		2.6	4.5	6.0	8.5	2.6	4.5	6.0	8.5
Bio-1	9.72	–	–	–	–	–	–	–	–
Bio-2	10.05	–	–	–	–	–	–	–	–
Bio-3	–	29.16	28.44	26.15	35.86	30.90	25.27	29.65	30.33
Bio-4	–	18.05	8.93	10.84	12.85	11.97	12.92	14.02	12.21
Bio-5	8.57	11.00	–	–	6.93	7.43	6.22	3.57	9.06
Bio-6	17.06	–	12.09	11.38	12.02	10.03	10.65	2.89	3.60
Bio-7	–	4.10	11.67	10.89	7.69	9.75	7.27	11.18	3.36
Bio-8	–	3.59	6.03	7.91	6.66	10.61	8.79	7.83	6.39
Bio-9	6.20	–	–	–	–	–	–	–	–
Bio-13	4.42	10.97	8.55	6.50	9.76	8.30	6.29	8.68	8.93
Bio-15	9.46	–	–	–	–	–	–	–	–
Bio-16	3.68	9.48	9.77	6.16	8.24	11.01	5.99	9.61	9.25
Bio-17	11.28	13.66	–	–	–	–	–	–	–
Bio-19	19.57	–	14.52	20.16	–	–	16.60	12.57	16.85

### Response curves of environmental gradients

**Fig 2:**
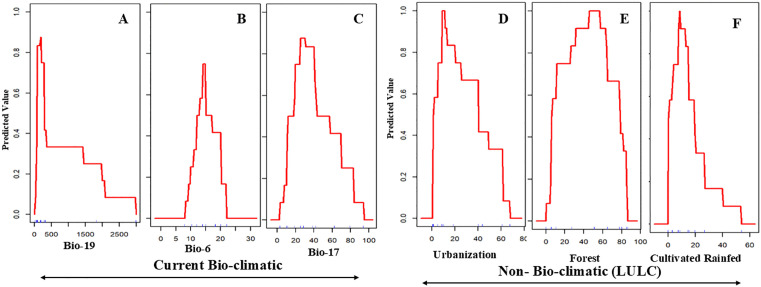
Response curves with most important variables of current bio-climatic (A to C) and non-bioclimatic variables (D to F) showing threshold levels for avocado suitability.

**Fig 3:**
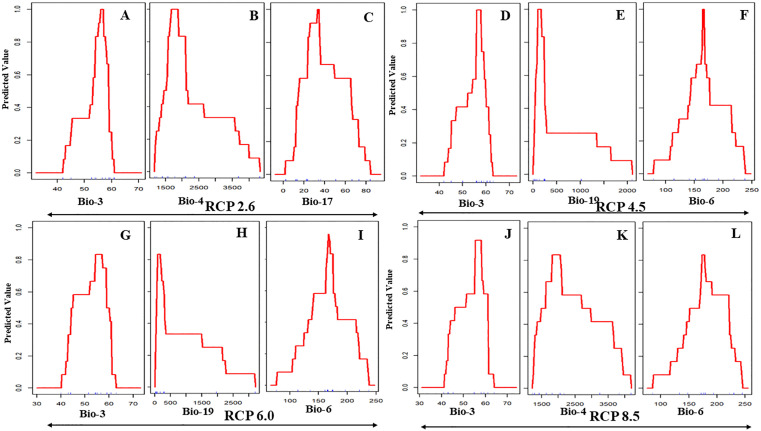
ROC curves showing threshold levels of avocado suitability with different 2050 bio-climatic time frame and four greenhouse gas projections (A to L).

**Fig 4:**
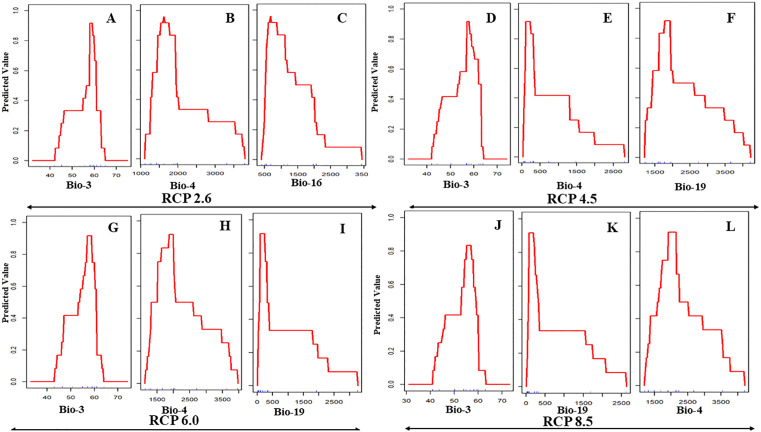
ROC curves showing threshold levels of avocado suitability with different 2070 bio-climatic time frame and four greenhouse gas projections (A to L).

Under current climatic conditions (Fig 2A–2C), avocado suitability is primarily shaped by Bio-19 (precipitation of the coldest quarter; optimum <500 mm, indicating preference for arid winters), Bio-6 (minimum temperature of the coldest month; optimum 10–15 °C, reflecting sensitivity to both frost and excessive heat), and Bio-17 (precipitation of the driest quarter; optimum 25–40 mm, indicating a requirement for moderate dry-season rainfall). Non-bioclimatic variables also exert considerable influence. Suitability peaks under urbanization levels of 20–40% (peri-urban agroforestry; Fig 2D), forest cover of 40–60% (semi-forested/agroforestry mosaics; Fig 2E), and rainfed cultivation of 30–40% (Fig 2F), with steep declines under higher intensities of these land-use factors. By 2050, response curves (Fig 3A–3L) demonstrate significant shifts across RCPs. Bio-3 (isothermality) consistently emerges as the dominant predictor, with optimal values around 60–65% under RCP 2.6 and a narrowing tolerance under RCP 4.5. Bio-19 broadens its tolerance range, shifting from <500 mm under RCP 6.0 to up to 1000 mm under RCP 4.5, while Bio-6 exhibits an upward peak at 15–18 °C across scenarios. Under RCP 8.5, flattening of Bio-3 and Bio-4 (temperature seasonality) curves suggests increasing generalist adaptability, potentially reflecting phenotypic plasticity. By 2070 (Fig 4A–4L), these trends intensify. Bio-3 remains the central determinant (60–65%) but expands under RCP 4.5 and RCP 6.0, suggesting enhanced tolerance to variability. Bio-4 widens its response range, while Bio-19 shows divergent patterns—shifting toward >1000 mm under RCP 4.5 but restricted to <1000 mm under RCP 6.0 and RCP 8.5. Collectively, these patterns point to an ecological trajectory of broader climatic adaptability, but also greater dependence on hydrological stability.

### Area suitability

Under present bioclimatic conditions, avocado shows a relatively constrained optimal suitability area of 39.97 × 10^3^ km^2^ (39,970 km^2^). By 2050, projections differ by RCP: RCP 2.6 shows an increase in optimum area to 15.07 × 10⁴ km^2^ (150,700 km^2^) and RCP 8.5 shows an increase to 16.05 × 10⁴ km^2^ (160,500 km^2^) (Table 3). We note that an increase in “optimum area” under some 2050 RCP projections (including RCP 8.5) can coexist with increasing spatial fragmentation and instability. In other words, pockets of newly suitable climate (often at higher elevations or latitudes) may appear and raise the total area meeting the numeric “optimum” threshold, while the overall landscape simultaneously fragments into smaller, more isolated refugia with higher uncertainty (see uncertainty maps, S1 Fig.). Therefore, the apparent mid-century increase in optimum area under RCP 8.5 does not contradict the broader observation of reduced refugia stability and long-term vulnerability under high-emission scenarios; rather it highlights a transient redistribution (creation of new but spatially fragmented optimum patches) that may not be sustainable into later timeframes (2070) when low-suitability zones and fragmentation intensify.

**Table 3:** Habitat suitability areas of *P. americana* under four classes with different environmental gradients.

Environmental Gradients	Optimum	Moderate	Marginal	Low
Current Bio-climatic	39.97 x 10^3^	37.09 x 10^4^	88.34 x 10^4^	14.85 10^5^
NBC	68.93 x 10^2^	13.16 x 10^4^	56.85 x 10^4^	16.35 10^5^
2050 RCP 2.6	15.07 x 10^4^	42.82 x 10^4^	10.60 x 10^5^	19.60 10^5^
2050 RCP 4.5	12.48 x 10^4^	25.28 x 10^4^	94.98 x 10^4^	89.54 10^4^
2050 RCP 6.0	10.36 x 10^4^	28.80 x 10^4^	10.43 x 10^5^	13.55 10^5^
2050 RCP 8.5	16.05 x 10^4^	29.63 x 10^4^	11.84 x 10^5^	21.22 10^5^
2070 RCP 2.6	17.79 x 10^4^	37.02 x 10^4^	10.48 x 10^5^	23.44 10^5^
2070 RCP 4.5	13.52 x 10^4^	29.32 x 10^4^	11.76 x 10^5^	12.04 10^5^
2070 RCP 6.0	18.51 x 10^4^	31.14 x 10^4^	10.31 x 10^5^	22.29 10^5^
2070 RCP 8.5	14.95 x 10^4^	29.29 x 10^4^	11.51 x 10^5^	18.95 10^5^

### Spatial extension of *Persea americana* in India

**Fig 5:**
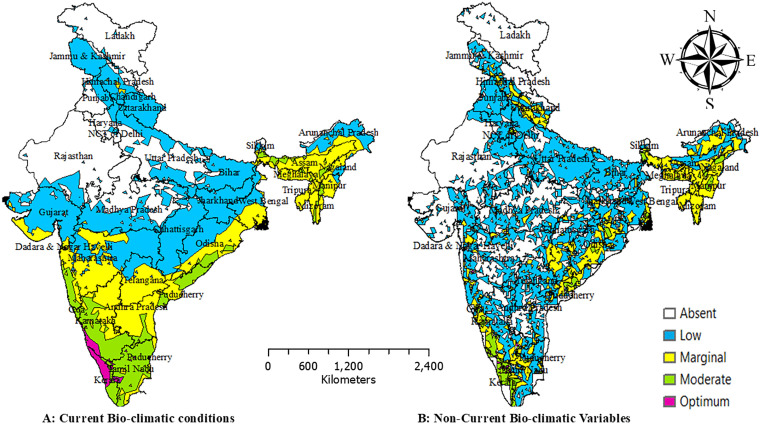
Habitat suitability of avocado with current (A) and non-bio-climatic variables (B). Maps created using ArcGIS software. Source: Esri. (2024). ArcGIS Desktop: Release 10.8. Redlands, California, United States: Environmental Systems Research Institute.

**Fig 6:**
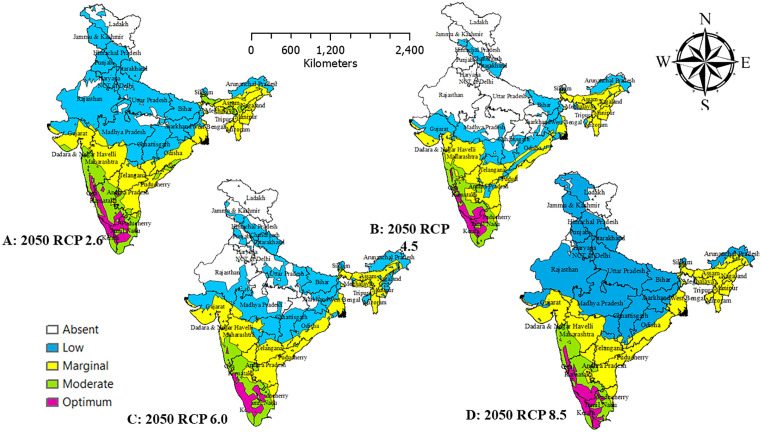
Habitat suitability of avocado with 2050 bio-climatic time frame and four greenhouse gas projections. Maps created using ArcGIS software. Source: Esri. (2024). ArcGIS Desktop: Release 10.8. Redlands, California, United States: Environmental Systems Research Institute.

**Fig 7:**
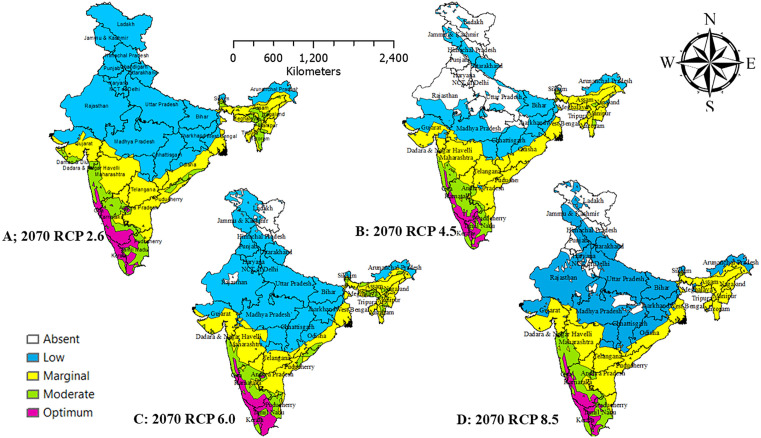
Habitat suitability of avocado with 2050 bio-climatic time frame and four greenhouse gas projections. Maps created using ArcGIS software. Source: Esri. (2024). ArcGIS Desktop: Release 10.8. Redlands, California, United States: Environmental Systems Research Institute.

Under current bioclimatic conditions (Fig 5A), most northern, northwestern, and central states (Rajasthan, Punjab, Haryana, Gujarat, Madhya Pradesh, Uttar Pradesh) fall within low-suitability zones. The eastern states (West Bengal, Odisha) exhibit marginal suitability, while the southern states (Karnataka, Andhra Pradesh, Tamil Nadu) support moderate suitability. Optimum suitability is restricted to small pockets in Kerala and Tamil Nadu, which function as present-day climatic refugia. The non-bioclimatic projections (Fig 5B) classify much of India as low suitability, yet broader marginal and moderate zones emerge in the south (Karnataka, Tamil Nadu, Kerala), east (West Bengal, Odisha), and northeast (Assam, Meghalaya, Nagaland, Tripura). Optimum habitats remain limited to Kerala and Tamil Nadu. By 2050, RCP 2.6 (Fig 6A) is the most favourable scenario, with moderate-to-optimum zones expanding across Kerala, Tamil Nadu, Karnataka, Maharashtra, and Telangana, while central and northern India remain largely unsuitable. Under RCP 4.5 (Fig 6B), southern suitability persists with minor losses, and modest gains appear in the eastern states (Assam, Tripura, Nagaland). RCP 6.0 (Fig 6C) results in widespread declines, shifting much of central and western India into marginal or poor categories, although small favourable pockets persist in the south. RCP 8.5 (Fig 6D) is the most detrimental, confining optimum suitability almost entirely to Kerala and Tamil Nadu, while much of northern, central, and western India becomes unsuitable. By 2070, overall suitability contracts further. RCP 2.6 (Fig 7A) maintains moderate-to-optimum zones in Kerala, Karnataka, Tamil Nadu, Odisha, and Telangana, but central and northern India remain marginal-to-poor. Under RCP 4.5 (Fig 7B), optimum habitats shrink to the southern Western Ghats, with marginal zones modestly expanding in the east. RCP 6.0 (Fig 7C) produces fragmentation and widespread habitat degradation, while RCP 8.5 (Fig 7D) restricts optimum suitability almost entirely to Kerala and southern Tamil Nadu, leaving most of India dominated by marginal or low-suitability zones under severe climate stress.

### Percent changes

Table 4 summarizes the percentage variation in avocado-suitable regions across four suitability classes—Optimum, Moderate, Marginal, and Low—under future climate scenarios (RCP 2.6, 4.5, 6.0, and 8.5 for both 2050 and 2070), relative to present bioclimatic conditions. A scenario based solely on non-bioclimatic (NBC) factors such as soil and topography is also included. The NBC scenario shows a substantial reduction in highly suitable zones, with Optimum suitability declining by 82.75% and Moderate suitability by 64.40%. Marginal suitability decreases by –35.64%, while Low suitability increases slightly by +10.15%. This pattern highlights a marked deterioration of potential avocado habitat when only edaphic and static environmental parameters are considered, without climatic drivers. In 2050, RCP 2.6 emerges as the most favourable scenario, with an increase of +277.22% in Optimum suitability, alongside gains in Moderate (+15.47%), Marginal (+20.02%), and Low (+32%) classes. This suggests that a low-emission trajectory could enhance ecosystems ideal for avocado cultivation. RCP 4.5 and RCP 6.0 also show notable increases in Optimum suitability (+212.42% and +159.38%, respectively), though both exhibit declines in Moderate suitability, while RCP 4.5 records a marked loss in Low suitability (–39.70%).

**Table 4:** Percent changes in area of *P. americana* with different temporal bio-climatic- GHG projections and non-bioclimatic variable in comparison to current bio-climatic variables.

Environmental Gradients	Optimum	Moderate	Marginal	Low
NBC	−82.75	−64.4	−35.64	10.15
2050 RCP 2.6	277.22	15.47	20.02	32.0
2050 RCP 4.5	212.42	−31.81	7.51	−39.70
2050 RCP 6.0	159.38	−22.35	18.07	−8.73
2050 RCP 8.5	301.50	−20.10	34.05	42.95
2070 RCP 2.6	345.26	−0.18	18.64	57.84
2070 RCP 4.5	238.33	−20.94	33.19	−18.90
2070 RCP 6.0	363.04	−16.02	16.74	50.12
2070 RCP 8.5	274.03	−21.02	30.36	27.64

These results indicate a redistribution of areas from less favourable to more climatically suitable zones. RCP 8.5 produces the largest relative increase in Optimum suitability (+301.50%) with favourable changes across other classes, suggesting the creation of new suitable zones at higher altitudes or latitudes, although such gains may not be stable in the long term. By 2070, the expansion of Optimum suitability continues, with the largest increases recorded under RCP 6.0 (+363.04%) and RCP 2.6 (+345.26%), indicating a long-term enhancement of highly suitable avocado habitats under moderate warming. These gains are coupled with declines in Moderate suitability, reflecting a transition of many areas from Moderate to Optimum status. RCP 8.5 also shows a substantial increase in Optimum suitability (+274.03%), though lower than under milder scenarios, suggesting that excessive warming begins to limit expansion potential. Across all RCPs for 2070, Low suitability zones expand, with the largest increase observed under RCP 2.6 (+57.84%), suggesting that avocado distribution may extend into ecologically marginal or transitional zones under future climates.

### Percent indigenous

Table 5 presents the Percent Indigenous Index (PI) for avocado across habitat suitability classes (Optimum, Moderate, Marginal, and Low) under different climatic and temporal scenarios. The index is derived by multiplying the area of each suitability class by the number of polygons and dividing by the total area (km^2^). Values range from 0 to 1, where lower values (~0) indicate that the distribution is ecologically restricted across suitability classes (i.e., concentrated in few suitability polygons or a narrow area (restricted, specialized distribution), and higher values (~1) indicate broader generality (widespread, generalist distribution). Under current bioclimatic conditions, avocado exhibits extremely low PI values, with scores of 0.03 in optimum habitats, 0.01 in moderate habitats, and 0.00 in both marginal and low suitability zones. This pattern reflects a highly restricted distribution, with the species confined to narrow climatic niches. By contrast, the non-bioclimatic (NBC) scenario, which incorporates soil, land use/land cover (LULC), and developmental threat index (DTI), reveals a different trend. PI values increase to 0.39 in optimum habitats, 0.09 in moderate zones, and 0.03 in marginal areas, indicating that avocado distribution becomes more widespread when shaped by edaphic and anthropogenic influences rather than climatic variables alone.

**Table 5:** Percent indigenous of *P. americana* under four suitability classes with different environmental gradients.

Environmental Gradients	Optimum	Moderate	Marginal	Low
Current	0.03	0.01	0.00	0.00
NBC	0.39	0.09	0.03	0.00
2050 RCP 2.6	0.01	0.01	0.00	0.00
2050 RCP 4.5	0.01	0.01	0.00	0.00
2050 RCP 6.0	0.01	0.00	0.00	0.00
2050 RCP 8.5	0.01	0.01	0.00	0.00
2070 RCP 2.6	0.00	0.01	0.00	0.00
2070 RCP 4.5	0.01	0.01	0.00	0.00
2070 RCP 6.0	0.00	0.01	0.00	0.00
2070 RCP 8.5	0.01	0.01	0.00	0.00

Future projections for 2050 and 2070 across all Representative Concentration Pathways (RCPs 2.6, 4.5, 6.0, and 8.5) consistently show low PI values, typically between 0.00 and 0.01 across all suitability classes. This persistent pattern suggests that avocado retains its specialized ecological characteristics under future climates, with minimal evidence of generalization or niche expansion. In several cases, PI values decrease to 0.00, further underscoring the species’ dependence on specific climatic conditions and its vulnerability to environmental change.

### Niche centroid value and hypervolume

**Fig 8:**
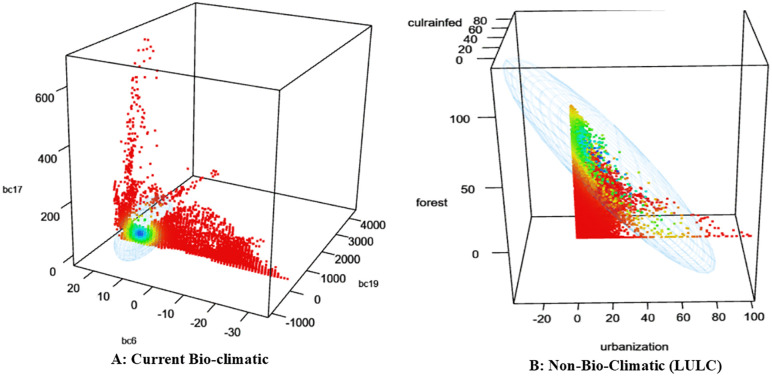
Niche hypervolume of avocado with current (A) and non-bio-climatic variables, specifically with LULC (B).

Table 6 presents the niche centroid values and hypervolumes of avocado across bioclimatic variables and climate change scenarios (RCPs) for 2050 and 2070. Centroid values reflect the mean environmental conditions of the species, while hypervolume represents the total ecological space occupied, with larger values indicating broader niche breadth. Under current conditions, centroid values were recorded for Bio-6 (14.73), Bio-17 (36.95), and Bio-19 (366.82), with a hypervolume of 4,670,780, reflecting a relatively restricted niche. These values are expressed in standardized environmental-units^3^ (dimensionless space derived from z-score–normalized predictors). Because absolute hypervolume magnitudes depend on the scaling of variables and the number of axes used, they are most meaningful when interpreted as relative changes (percent expansion or contraction) with respect to the present baseline. Accordingly, percent-change values have been added in parentheses for clarity. Relative to this baseline, RCP projections showed changes ranging from contraction under RCP 2.6 in 2050 (2,922,868; –37.4%) to major expansions exceeding 122 million under RCP 6.0 in 2070 (+2516%). In 2050, RCP 6.0 recorded the largest expansion (12,364,779; + 165%), with centroid shifts in Bio-4 (164.26) and Bio-19 (358.82), indicating entry into novel climatic zones. By contrast, RCP 2.6 exhibited a moderate contraction of the niche volume relative to the present baseline, reflecting limited expansion potential under mild warming. By 2070, niche expansion intensified markedly, with hypervolumes of 98,730,089 (+2014%) under RCP 4.5 and 122,142,908 (+2516%) under RCP 6.0, accompanied by elevated centroid values for Bio-4 (Temperature Seasonality) and Bio-19 (Precipitation of the Coldest Quarter). These shifts indicate a pronounced broadening of the avocado climatic niche and displacement into novel environmental space, implying strong ecological plasticity under sustained warming. RCP 8.5 also demonstrated substantial expansion (78,597,673; + 1582%), though accompanied by higher climatic instability and potential fragmentation of suitable zones. Collectively, these results indicate potential niche expansion up to ~25-fold relative to current conditions. Independent analysis of land-use/land-cover (LULC) variables (urbanization, rainfed agriculture, forest) produced a much smaller hypervolume (346,566.8), confirming that climate is the dominant factor shaping the avocado niche. However, conversion to urban land (22.73%) and cultivated land (10.89%) may still exert localized effects on distribution. Fig 8A and 8B illustrate these contrasts: under current bioclimatic conditions, avocado exhibits a condensed niche clustered around Bio-6, Bio-17, and Bio-19, reflecting niche conservatism. Under LULC conditions, the niche broadens and becomes irregular, suggesting greater ecological flexibility in managed landscapes.

**Table 6:** Niche centroid value and hypervolume of *P. americana.* The reported hypervolume values represent the multidimensional ecological space occupied by *Persea americana* based on the three most influential bioclimatic predictors. These values are expressed in standardized environmental-units^3^ (dimensionless space derived from z-score–normalized predictors).

Bio-Climatic Variables		Bio-3	Bio-4	Bio-6	Bio-16	Bio-17	Bio-19	Hypervolume
Current Bio-Climatic		–	–	14.73	–	36.95	366.82	4670780
2050 RCPs	2.6	55.54	1885.7	–	–	37.77	–	2922868
	4.5	55.47	–	165.3	–	–	249.47	5483758
	6	53.17	–	164.26	–	–	358.82	12364779
	8.5	54.36	2203.09	183.59	–	–	–	5084607
2070 RCPs	2.6	57.78	1836.95	–	1111.21	–	–	12169020
	4.5	56.27	2143.09	–	–	–	327.63	98730089
	6	55.71	2107.91	–	–	–	399.34	122142908
	8.5	54.18	2238.54	–	–	–	267.63	78597673

**Fig 9:**
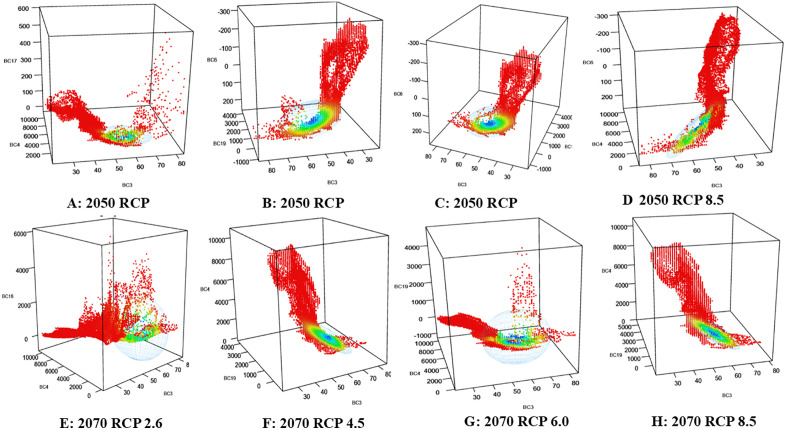
Niche hypervolume showing fundamental and realized niche distribution of avocado with 2050 and 2070 bio-climatic time frames and their respective greenhouse gas projections (A to H).

Future projections reinforce these trends. In 2050, RCPs 2.6 and 4.5 (Fig 9A and 9B) show modest expansions concentrated along Bio-19, while RCPs 6.0 and 8.5 (Fig 9C and 9D) reveal greater dispersion along Bio-4 and Bio-6, indicating adaptation to more humid and temperate environments. By 2070, expansion intensifies: RCPs 2.6 and 4.5 (Fig 9E and 9F) show moderate growth, RCP 6.0 (Fig 9G) exhibits the broadest hypervolume with significant dispersion, and RCP 8.5 (Fig 9H) expands vertically along Bio-19 and Bio-6, suggesting enhanced precipitation tolerance and reduced climatic specificity. Overall, the results reveal dual ecological behaviour: a restricted climatic niche under present conditions, coupled with the potential for broad niche expansion under future scenarios. This indicates both vulnerability and adaptive potential, with climatic variables exerting stronger influence than land-use factors.

## Discussion

### Model performance

The reliability of SDM predictions depends critically on both the accuracy and independence of occurrence records. Large datasets with spatial errors or high spatial autocorrelation can bias model outcomes and artificially inflate predictive performance, whereas smaller but high-quality, spatially independent datasets often yield more ecologically meaningful predictions. Several studies have shown that SDMs can remain reliable even with limited sample sizes, provided that occurrence points are precise and not spatially clustered, thereby reducing overfitting and improving transferability. In this context, the relatively small dataset of 35 occurrence records used in the present study remains suitable, as emphasis was placed on ensuring data accuracy and minimizing spatial autocorrelation rather than merely maximizing sample size. To mitigate potential spatial and environmental bias, a 25 km^2^ spatial thinning filter was applied, regularization tuning for MaxEnt was implemented using ENMeval, and highly correlated predictors (r ≥ 0.85; VIF < 10) were excluded. Cross-validation and AUC-weighted ensemble averaging further reduced the influence of overfitting, providing a balanced representation of potential suitability despite the modest sample size. While small datasets inherently limit the resolution of local-scale inference, these methodological safeguards ensure that model predictions remain ecologically robust and transferable.

We compare our findings with a small but growing body of ENM literature on avocado and related agro-ecological assessments. Sivaraj et al. [26] provided an early, valuable MaxEnt-based climatic envelope for southern India, identifying temperature and precipitation thresholds restricting avocado to southern hill and coastal pockets. Our results are broadly consistent with those spatial patterns (concentration of optimum suitability in the southern Western Ghats and parts of Tamil Nadu and Kerala), but we find additional nuance: by integrating non-bioclimatic predictors (soil depth, land-use, and habitat heterogeneity) and using an ensemble approach, our models reveal that many climatically suitable areas are rendered marginal or unsuitable once edaphic and anthropogenic constraints are considered. This complements regional reports where ENM-guided zoning increased yields by focusing on elevation-specific cultivation (e.g., Colombian Andean studies) and where careful selection of cultivation zones in Kenya’s Rift Valley facilitated export growth. Thus, while climate envelopes identify potential climatic space, our integrated ESDM highlights the critical influence of local landscape and soil constraints, supporting the notion that multi-factor suitability assessments better inform on-ground decision making than climate-only projections.

Sensitivity values remain consistently high across all scenarios (0.83–0.87), reflecting the model’s strong ability to correctly identify presence locations. Specificity also demonstrates robust performance, ranging from 0.82 under current conditions to a maximum of 0.90 in the 2070 RCP 6.0 scenario, indicating efficient exclusion of unsuitable habitats [56]. The elevated specificity observed in 2070 RCP 6.0 suggests a possible improvement in model accuracy as climate extremes intensify. Kappa values, which measure agreement beyond chance, increase from 0.48 under present conditions to 0.59 in the 2070 RCP 6.0 scenario, indicating moderate to substantial agreement. Similarly, the True Skill Statistic (TSS), which integrates sensitivity and specificity, rises from 0.67 at present to a peak of 0.75 in the 2050 RCP 2.6 scenario, highlighting improved predictive accuracy under mitigation-oriented climate pathways. Models under high-emission pathways (RCP 8.5) typically yield slightly lower but still reliable evaluation scores compared to lower RCPs, suggesting that while uncertainty increases under severe climate conditions, ensemble approaches preserve substantial predictive power [57].

This robustness underscores the importance of ensemble methodologies, which integrate the strengths of multiple algorithms and reduce the biases inherent in individual models [58]. The consistently strong performance of the ESDM across different temporal and emission scenarios therefore highlights the effectiveness of ensemble approaches for anticipating species distribution shifts under climate change, reinforcing their value for proactive biodiversity conservation and climate adaptation strategies [59].

### Climatic and non-climatic variables importance

The analysis of variable importance across current and projected scenarios reveals substantial ecological shifts influencing avocado suitability in India (Tables 2–4; Fig 2 to Fig 7). Under present conditions, precipitation of the coldest quarter (BIO-19), minimum temperature of the coldest month (BIO-6), and precipitation of the driest quarter (BIO-17) emerge as dominant predictors. These variables underscore avocado’s sensitivity to cold-season moisture availability and frost thresholds that are critical for flowering and early fruit development. By mid-century (2050), however, all RCPs indicate a marked shift, with isothermality (BIO-3) becoming the leading predictor, peaking at 35.86% under RCP 8.5. This highlights the increasing importance of thermal stability—reflecting the balance between daily and seasonal temperature variation—in shaping future avocado suitability. Refugia is therefore likely to persist in regions that buffer seasonal extremes. Moderate increases in the importance of temperature seasonality (BIO-4, BIO-7) further suggest a transition from fixed cold thresholds to broader tolerance of climatic variability, consistent with global observations [60,61].

The supplementary assessment of non-bioclimatic factors underscores the significance of anthropogenic and landscape-level drivers. Urbanization (importance > 22) emerges as the strongest constraint, aligning with findings from peri-urban Mexico [62]. Land-cover variables (cultivated and forest) enhance suitability by supporting pollination services and regulating microclimate, while soil attributes—such as bulk density, clay content, and water-holding capacity—exert moderate influence, consistent with agronomic preferences for well-structured loams [63]. Terrain variables (elevation, slope, aspect) and vegetation vigour indices (NDVI, shrub density) also contribute meaningfully, reflecting avocado’s affinity for mid-altitude, moderately sloping landscapes with abundant green biomass [64]. In contrast, road density and cultivation intensity, though less influential, highlight potential risks associated with human pressure [65].

Collectively, these findings emphasize the need for integrated agro-ecological planning. Priority actions include conserving forest buffers in the Western Ghats and northeastern India, safeguarding cool-humid niches at higher altitudes, and adopting management interventions such as shading, mulching, and water harvesting to stabilize isothermality and soil moisture. Equally critical will be restricting urban encroachment and preserving loamy soils in mid-altitude zones to ensure resilient avocado supply chains under accelerating climate change.

### Response curves of environmental gradients

Response curves indicate that avocado functions as a specialist of moderately dry and thermally stable climates, with optimal suitability linked to minimal winter precipitation (BIO-19), moderate cold-month minima (BIO-6), and adequate dry-season moisture (BIO-17). These constraints restrict core habitat to the semi-arid forests of the southern Western Ghats and Deccan Plateau [55,66]. The steep response of BIO-6 underscores avocado’s sensitivity to thermal thresholds: even minor warming in coastal lowlands or urban heat islands may exceed stress tolerance, while increasing aridity under RCP 8.5 could surpass BIO-19 limits.

These patterns reflect narrow physiological tolerances, where extreme cold impedes germination and metabolism, and excessive rainfall during dormancy fosters pathogen infections [66,67]. Non-climatic response curves reinforce this vulnerability, showing preferences for semi-natural mosaics and moderate forest cover, but low tolerance for urbanization and intensive agriculture. Consequently, deforestation and land-use intensification remain critical threats, highlighting the importance of community forestry, buffer zones, and agroforestry corridors [68,69].

Future projections indicate persistence of these climatic sensitivities. By 2050, BIO-6 remains the dominant predictor across all RCPs, confirming thermal-niche conservatism with optima clustered between 16–18 °C. Under RCP 8.5, declines in isothermality (BIO-3) coupled with variable BIO-4/BIO-19 responses suggest partial drought adaptation but continued vulnerability to shifting rainfall regimes. Suitability contracts into micro-refugia where cold-month minima remain stable and moderate drought persists [70,71]. By 2070, BIO-3 becomes the primary constraint, with optimal suitability narrowed to a 58–60% range, while BIO-16/19 continue to favour moderate-to-low rainfall conditions. Compared to 2050, response peaks shift minimally, but curve flattening and compression under RCP 6.0 and RCP 8.5 reflect progressive habitat degradation, niche contraction, and declining resilience.

### Area suitability

The analysis reveals substantial shifts in the spatial suitability of avocado cultivation under different climate scenarios. Under present conditions, most areas fall within marginal or low suitability zones, likely reflecting prevailing temperature and precipitation constraints. However, under future RCP scenarios—particularly RCP 2.6 and RCP 6.0—the extent of optimally suitable areas is projected to expand considerably. This indicates that avocado may benefit from moderate warming and associated climatic changes.

Avocado generally thrives within specific thresholds of 15–25 °C temperature and 1,000–2,000 mm annual precipitation. Accordingly, slight temperature increases may enhance suitability in cooler regions, while extreme warming, as projected under RCP 8.5, could impose heat stress and reduce suitability in tropical and lowland areas. Although some climate projections suggest enhanced suitability in new regions, these potential gains may be offset by heightened evapotranspiration, altered phenology, and increased risks of disease susceptibility (e.g., root rot from *Phytophthora cinnamomi*).

### Spatial extension of *Persea americana* in India

Current bioclimatic conditions indicate a limited ecological range for avocado, with suitability largely confined to warm, humid climates in southern India. Northern and northwestern regions remain unsuitable due to seasonal temperature extremes, aridity, and low annual rainfall. Optimal and moderately suitable habitats align with humid to semi-humid zones, particularly the Western Ghats, which provide stable temperatures and consistent precipitation. These findings are consistent with distribution modelling studies in tropical India, which show thermophilic and mesic species concentrated in the Western and Eastern Ghats under favourable precipitation and mean annual temperature regimes [72,73]. Climate envelope projections further suggest that shifts in rainfall and temperature will progressively relocate suitable zones southward, reducing the northern habitat boundary [74]. Thus, moderate suitability areas may become marginal, and optimal zones may contract, underscoring the importance of conservation in southern India, particularly the Western Ghats, which function as climatic refugia [75].

It is important to note that the numerical increase in optimum habitat area under RCP 8.5 (as shown in Table 3) does not imply ecological expansion or long-term stability. Rather, it reflects a transient redistribution of suitable conditions into new, spatially fragmented zones—typically at higher elevations or latitudes—as warming intensifies. These gains are accompanied by substantial losses in current refugia and a rise in climatic uncertainty. By 2070, many of these newly formed patches are predicted to degrade or disappear, resulting in an overall contraction of stable habitat. This interpretation reconciles the apparent discrepancy between the tabulated area increases and the narrative of reduced refugia stability under RCP 8.5. In essence, the total “optimum area” expands numerically but becomes increasingly fragmented and ecologically unstable, highlighting the need for caution when interpreting surface-area gains without considering spatial configuration and persistence over time.

A potential bias arises from uneven sampling: most occurrence records derive from southern India where avocado is cultivated, while northern and central regions remain underrepresented. This may underestimate the species’ potential in climatically favourable but poorly surveyed regions, leading to projections that overemphasize southern suitability. Future validation should involve broader occurrence datasets, targeted field surveys, and experimental trials to refine model transferability. Projected changes highlight habitat reduction and fragmentation, with moderate and optimal zones declining and marginal zones expanding, particularly in central and eastern India. Persistence of suitability in Kerala and Tamil Nadu reflects the buffering role of the Western Ghats [75], while rising precipitation may increase marginal suitability in the northeast [76]. These patterns point to the need for proactive conservation in southern and northeastern India, alongside monitoring of at-risk central and northern areas. Comparisons of RCPs for 2050 show progressive habitat loss with higher emissions, reflecting reliance on moderate temperature (15–25 °C), humidity, and stable rainfall—factors threatened by climate change [77]. Southern India, especially the Western Ghats, consistently maintains suitability due to orographic rainfall, elevation, and microclimatic buffering [78]. In contrast, northern and central plains transition from marginal to unsuitable under higher RCPs, while the northeast may gain importance as a niche under lower RCPs due to rainfall increases and canopy cover [79].

These results highlight the urgency of climate-adaptive interventions, including designation of priority conservation areas, agroforestry with resilient avocado varieties, and land-use planning to sustain ecosystem connectivity. Integrating in-situ conservation with assisted migration and buffer-zone restoration may strengthen resilience [55]. Low-emission pathways (RCP 2.6) remain critical to preserve current zones and slow contraction of the crop’s range. By 2070, all RCPs indicate further contraction—particularly under RCP 4.5, 6.0, and 8.5—with suitability retreating to climatically stable southern refugia. The Western Ghats emerge as consistent refugia due to elevation gradients, reliable rainfall, and thermal buffering. Central and northern India shift predominantly to marginal or unsuitable conditions, while northeastern suitability remains limited by rainfall variability and soil constraints. Overall, avocado’s future distribution is projected to contract southwards, reflecting ecological vulnerability and emphasizing the need for spatially focused conservation and adaptation strategies in southern and eastern India [80–82].

### Percent changes in suitability areas

The comparative analysis of percentage changes in avocado-suitable areas across RCP-based bioclimatic forecasts and non-bioclimatic predictors underscores the dominant role of climate in shaping habitat suitability in India (Table 2–4; Fig 2 to Fig 7). Models relying only on non-bioclimatic (NBC) variables show sharp declines in Optimum (−82.75%) and Moderate (−64.4%) zones, reflecting the limited explanatory capacity of static factors such as soil or terrain, consistent with [57]. By contrast, RCP-based projections for 2050 and 2070 generally indicate significant expansions of Optimum zones. In 2050, RCP 8.5 (+301.50%) and RCP 2.6 (+277.22%) show the largest gains, suggesting that warming up to a threshold may create new thermally suitable areas at higher elevations and latitudes, in line with global findings on thermophilic crops. This trend continues by 2070, with maximum increases under RCP 6.0 (+363.04%) and RCP 2.6 (+345.26%), reinforcing the long-term importance of southern and topographically buffered regions. Moderate suitability declines in most scenarios, most notably under RCP 4.5 in 2050 (−31.81%). Marginal and Low zones show mixed patterns, with RCP 8.5 in 2050 producing the greatest increases (Marginal +34.05%; Low + 42.95%), suggesting range extension into presently unsuitable areas that remain suboptimal for productive cultivation. By 2070, Low zones expand further, peaking under RCP 2.6 (+57.84%), reflecting climate-driven niche erosion and persistence in marginal habitats, as also noted by [83].

Overall, these results indicate a contraction of avocado’s core niche towards climatically stable refugia such as the Western Ghats, while central and northern India shift to marginal or unsuitable categories due to rising temperatures and erratic precipitation.

### Percent indigenous

Although avocado is strongly shaped by climatic factors, non-climatic variables can modify its distribution and promote more generalist behavior [20,65]. The disparity between low climatic and high NBC-based index values underscores the importance of integrating multiple environmental layers in modeling. This suggests that while climate change may not substantially expand the niche, anthropogenic changes such as land conversion, altered soil profiles, and habitat disruption could facilitate dissemination. Currently, avocado exhibits native-like behavior under prevailing and projected climates, reflecting ecological stability and niche conservatism. However, under the influence of non-climatic drivers, the species shows increased generality, particularly in optimal zones. Conservation and habitat management strategies must therefore integrate land-use regulation and soil management alongside climate adaptation.

### Niche centroid value and hypervolume

Comparisons highlight BIO-4 (precipitation of the driest month) as the dominant driver of niche development, particularly under high-emission scenarios (RCP 6.0 and RCP 8.5 in 2070), while BIO-3 (isothermality) and BIO-17 (precipitation of the driest quarter) stabilize niche preservation. Rising hypervolume values across future RCPs indicate niche expansion and environmental generalization, likely linked to increased dry-season precipitation and shifts in climatic stability, consistent with [84]. Comparative hypervolume analysis (Table 6; Fig 8, Fig 9A to Fig 9H) shows compact, stable niches under low-emission pathways (RCP 2.6), whereas higher emissions (RCP 4.5, 6.0, 8.5) drive fragmentation and dispersal, reflecting reduced suitability and ecological stress.

These findings echo earlier studies demonstrating climate-driven niche shifts, habitat loss, and heightened vulnerability [85]. Observed niche alterations underscore the urgency of preemptive conservation, including preservation of core habitats, restoration of landscape connectivity, and mitigation of anthropogenic stressors. Integrating climate projections into conservation and agricultural planning is essential for sustaining avocado cultivation and biodiversity resilience.

### Policy and sustainability implications

Expanding avocado cultivation into newly suitable regions offers economic potential. India currently imports avocados worth ~INR 120–150 crores annually [15], and domestic expansion into climatically suitable eastern and northeastern zones could reduce import dependence and diversify farmer incomes. Evidence from Colombia and Kenya shows that climate- and ENM-guided zoning can increase production by 20–30% [20,25], highlighting potential gains for Indian smallholders. Yet, expansion raises ecological and ethical concerns. Experiences from Mexico reveal risks of deforestation, biodiversity loss, and water overuse linked to unregulated avocado growth [62]. In India, safeguarding the Western Ghats and northeastern forests is critical. Agroforestry-based integration of avocado at forest–agriculture interfaces offer a sustainable pathway, aligning commercial expansion with ecosystem conservation. This approach can buffer climate risks, enhance pollinator habitats, and strengthen rural livelihoods, balancing production growth with ecological stewardship.

## Conclusion

This study represents the first spatially explicit ensemble modelling assessment of *Persea americana* (avocado) suitability across India under current and future climatic conditions. By integrating bioclimatic, edaphic, topographic, and land-use variables, we delineated areas of potential expansion, contraction, and persistence under different Representative Concentration Pathways (RCPs). Our results indicate that under low- to moderate-emission scenarios (RCP 2.6 and RCP 6.0), avocado suitability is projected to expand modestly, primarily along southern and eastern highlands where temperature and precipitation regimes remain within optimal thresholds. These scenarios reflect conditions favourable for targeted expansion through agroforestry and diversification programmes.

Under high-emission scenarios (RCP 8.5), however, the apparent numerical increase in optimum area represents transient redistribution into fragmented, high-elevation zones rather than true ecological expansion. By 2070, these fragmented patches are predicted to degrade or disappear, resulting in overall contraction and instability of suitable habitats. This finding reconciles the earlier inconsistency between tabulated figures and the broader narrative of declining refugia stability under climate stress.

Model outputs collectively highlight the Western Ghats, parts of Kerala and Tamil Nadu, and isolated pockets of the northeastern hill states as persistent climatic refugia. These zones should be prioritized for conservation, adaptive agroforestry, and cultivar improvement efforts. Conversely, central and northern India are likely to experience progressive suitability loss, emphasizing the importance of low-emission development trajectories to sustain ecological and production resilience.

Future studies with expanded occurrence datasets and refined validation across underrepresented regions will help reduce spatial bias and strengthen predictive robustness. The present findings provide a reproducible spatial framework for guiding site-specific planning, varietal introduction, and land-use policy for climate-resilient avocado development in India.

## Supporting information

S1 Table Details of different bioclimatic variables and GPS coordinates of avocado growing locations. (DOC)

S2 Table Performance of individual algorithms utilized for ESDM with current bio-climatic variables. (DOCX)

S3 Table Performance of individual algorithms utilized for ESDM with RCPs 2.6, 4.5, 6.0 and 8.5 with 2050 and 270 bio-climatic time-frames. (DOCX)

S4 Table Performance of individual algorithms utilized for ESDM with NBC. (DOCX)

S1 Fig Uncertainty Maps with different bioclimatic and non-bioclimatic predictors. Uncertainty maps with different bioclimatic and non-bioclimatic predictors. Maps created using ArcGIS software. Source: Esri. (2024). ArcGIS Desktop: Release 10.8. Redlands, California, United States: Environmental Systems Research Institute.
(TIF)

S2 Fig Variable Importance Percentage of different non bio-climatic variables related with LULC, plant community dynamics, soil, terrain, slopes and anthropogenic factors. (TIF)
