# Supplementary material for: Climate-driven shifts in avocado suitability zones in India: Insights from ensemble modelling and niche hypervolume
Source: PLoS One. 2026 Jan 14;21(1):e0338518. doi: 10.1371/journal.pone.0338518 (PMC12803459; doi:10.1371/journal.pone.0338518)
Supplement: S1 Table — (DOC) [file pone.0338518.s001.doc]

**Supplementary Tables**

Supplementary Table 1. Details of different bioclimatic variables and GPS Coordinates of Avocado Research Locations used in the study

| **Code** | **Environmental variables and their abbreviations** | **Scaling factor** | **Unit** |
| --- | --- | --- | --- |
| Bio-1 | Annual mean temperature (AMT) | 10 | ^o^C |
| Bio-2 | Mean diurnal range (MeDR) | 10 | ^o^C |
| Bio-3 | Isothermality (BC2/BC7) (× 100) (Iso) | 100 | - |
| Bio-4 | Temperature seasonality (standard deviation × 100) (TempS) | 100 | - |
| Bio-5 | Maximum temperature of warmest month (MaTWaM) | 10 | ^o^C |
| Bio-6 | Minimum temperature of coldest month (MiTCM) | 10 | ^o^C |
| Bio-7 | Temperature annual range (BC 5–BC 6) (TAR) | 10 | ^o^C |
| Bio-8 | Mean temperature of wettest quarter (MeTWeQ) | 10 | ^o^C |
| Bio-9 | Mean temperature of driest quarter (MeTDQ) | 10 | ^o^C |
| Bio-10 | Mean temperature of warmest quarter (MeTWaQ) | 10 | ^o^C |
| Bio-11 | Mean temperature of coldest quarter (MeTCQ) | 10 | ^o^C |
| Bio-12 | Annual precipitation (AnPr) | 1 | mm |
| Bio-13 | Precipitation of wettest month (PrWeM) | 1 | mm |
| Bio-14 | Precipitation of driest month (PrDM) | 100 | mm |
| Bio-15 | Precipitation seasonality (coefficient of variation) (PrS) | 1 | Fraction |
| Bio-16 | Precipitation of wettest quarter (PrWeQ) | 1 | mm |
| Bio-17 | Precipitation of driest quarter (PrDQ) | 1 | mm |
| Bio-18 | Precipitation of warmest quarter (PrWaQ) | 1 | mm |
| Bio-19 | Precipitation of coldest quarter (PrCQ) | 1 | mm |

GPS Coordinates of Avocado Research Locations

| Location | Latitude (DMS) | Longitude (DMS) | Latitude (Decimal) | Longitude (Decimal) | Elevation (m) |
| --- | --- | --- | --- | --- | --- |
| CCRI, CRS, Balehonnur, Karnataka | 13°34′00″ N | 75°48′00″ E | 13.5667 | 75.8000 | 750 |
| CRSS, Chettalli, Karnataka | 12.3702° N | 75.8309° E | 12.3702 | 75.8309 | 982.52 |
| RCRS, Chundale, Kerala | 11°00′09″ N | 76°00′00″ E | 11.0025 | 76.0000 | 840 |
| RCRS, Thandigudi, Tamil Nadu | 10°18′39″ N | 77°38′34″ E | 10.3108 | 77.6428 | 1130 |
| RCRS, R.V. Nagar, Andhra Pradesh | 17°52′45″ N | 82°14′30″ E | 17.8792 | 82.2417 | 838–997 |
| RCRS, Diphu, Assam | 25°55′12″ N | 93°44′00″ E | 25.9200 | 93.7333 | 170 |
| ICAR-IIHR-CHES, Chettalli, Karnataka | 12°26′00″ N | 75°47′00″ E | 12.4333 | 75.7833 | 945 |
| KVK, Ranipool, Sikkim | 27°17′06.5″ N | 88°35′29.7″ E | 27.2851 | 88.5916 | 980 |
