# Supplementary material for: Climate-driven shifts in avocado suitability zones in India: Insights from ensemble modelling and niche hypervolume
Source: PLoS One. 2026 Jan 14;21(1):e0338518. doi: 10.1371/journal.pone.0338518 (PMC12803459; doi:10.1371/journal.pone.0338518)
Supplement: S2 Table — (DOCX) [file pone.0338518.s002.docx]

Supplementary Table 2. Performance of individual algorithms utilized for ESDM with current bio-climatic variables

|  | AUC | Sensitivity | Specificity | Kappa | TSS |
| --- | --- | --- | --- | --- | --- |
| Current GLM | 0.91 | 0.88 | 0.85 | 0.23 | 0.73 |
| Current GAM | 0.85 | 0.83 | 0.85 | 0.24 | 0.68 |
| Current MARS | 0.87 | 0.83 | 0.86 | 0.27 | 0.69 |
| Current CTA | 0.83 | 0.83 | 0.84 | 0.67 | 0.67 |
| Current RF | 0.94 | 0.88 | 0.91 | 0.79 | 0.79 |
| Current ANN | 0.85 | 0.84 | 0.64 | 0.49 | 0.49 |
| Current SVM | 0.85 | 0.88 | 0.79 | 0.67 | 0.67 |
