# Supplementary material for: Climate-driven shifts in avocado suitability zones in India: Insights from ensemble modelling and niche hypervolume
Source: PLoS One. 2026 Jan 14;21(1):e0338518. doi: 10.1371/journal.pone.0338518 (PMC12803459; doi:10.1371/journal.pone.0338518)
Supplement: S3 Table — (DOCX) [file pone.0338518.s003.docx]

Supplementary Table 3. Performance of individual algorithms utilized for ESDM with RCPs 2.6, 4.5, 6.0 and 8.5 with 2050 and 270 bio-climatic time-frames

|  | 2050 Time Frame | | | | | 2070 Time Frame | | | | | |
| --- | --- | --- | --- | --- | --- | --- | --- | --- | --- | --- | --- |
| RCPs | Algorithm | AUC | Sensitivity | Specificity | Kappa | TSS | AUC | Sensitivity | Specificity | Kappa | TSS |
| RCP 2.6 | GLM | 0.91 | 0.89 | 0.89 | 0.31 | 0.78 | 0.86 | 0.80 | 0.86 | 0.31 | 0.66 |
|  | GAM | 0.89 | 0.86 | 0.88 | 0.30 | 0.73 | 0.85 | 0.84 | 0.84 | 0.24 | 0.68 |
|  | MARS | 0.90 | 0.81 | 0.94 | 0.45 | 0.76 | 0.86 | 0.80 | 0.94 | 0.46 | 0.74 |
|  | CTA | 0.84 | 0.85 | 0.83 | 0.68 | 0.68 | 0.79 | 0.83 | 0.76 | 0.59 | 0.59 |
|  | RF | 0.92 | 0.88 | 0.91 | 0.79 | 0.79 | 0.90 | 0.84 | 0.86 | 0.70 | 0.70 |
|  | ANN | 0.87 | 0.89 | 0.81 | 0.70 | 0.70 | 0.88 | 0.87 | 0.73 | 0.60 | 0.60 |
|  | SVM | 0.94 | 0.90 | 0.93 | 0.83 | 0.83 | 0.90 | 0.90 | 0.89 | 0.79 | 0.79 |
| RCP 4.5 | GLM | 0.88 | 0.78 | 0.87 | 0.24 | 0.65 | 0.88 | 0.82 | 0.81 | 0.19 | 0.63 |
|  | GAM | 0.91 | 0.85 | 0.93 | 0.42 | 0.77 | 0.91 | 0.87 | 0.86 | 0.26 | 0.73 |
|  | MARS | 0.88 | 0.79 | 0.95 | 0.50 | 0.74 | 0.87 | 0.76 | 0.94 | 0.39 | 0.70 |
|  | CTA | 0.80 | 0.78 | 0.83 | 0.60 | 0.60 | 0.87 | 0.84 | 0.89 | 0.73 | 0.73 |
|  | RF | 0.93 | 0.96 | 0.87 | 0.82 | 0.82 | 0.88 | 0.84 | 0.83 | 0.68 | 0.68 |
|  | ANN | 0.87 | 0.86 | 0.83 | 0.69 | 0.69 | 0.83 | 0.84 | 0.76 | 0.60 | 0.60 |
|  | SVM | 0.90 | 0.85 | 0.89 | 0.74 | 0.74 | 0.91 | 0.88 | 0.88 | 0.76 | 0.76 |
| RCP 6.0 | GLM | 0.89 | 0.83 | 0.87 | 0.28 | 0.70 | 0.91 | 0.84 | 0.89 | 0.32 | 0.73 |
|  | GAM | 0.88 | 0.86 | 0.88 | 0.31 | 0.74 | 0.84 | 0.74 | 0.93 | 0.40 | 0.67 |
|  | MARS | 0.86 | 0.78 | 0.92 | 0.34 | 0.70 | 0.84 | 0.74 | 0.92 | 0.42 | 0.67 |
|  | CTA | 0.84 | 0.85 | 0.83 | 0.68 | 0.68 | 0.85 | 0.81 | 0.90 | 0.71 | 0.71 |
|  | RF | 0.93 | 0.90 | 0.89 | 0.79 | 0.79 | 0.90 | 0.88 | 0.88 | 0.76 | 0.76 |
|  | ANN | 0.90 | 0.87 | 0.86 | 0.73 | 0.73 | 0.88 | 0.89 | 0.85 | 0.74 | 0.74 |
|  | SVM | 0.92 | 0.92 | 0.84 | 0.77 | 0.77 | 0.94 | 0.90 | 0.91 | 0.81 | 0.81 |
| RCP 8.5 | GLM | 0.90 | 0.86 | 0.87 | 0.30 | 0.73 | 0.88 | 0.87 | 0.82 | 0.20 | 0.69 |
|  | GAM | 0.89 | 0.83 | 0.88 | 0.35 | 0.71 | 0.88 | 0.82 | 0.93 | 0.47 | 0.75 |
|  | MARS | 0.88 | 0.80 | 0.97 | 0.57 | 0.77 | 0.87 | 0.76 | 0.92 | 0.35 | 0.68 |
|  | CTA | 0.81 | 0.81 | 0.82 | 0.63 | 0.63 | 0.82 | 0.89 | 0.76 | 0.65 | 0.65 |
|  | RF | 0.90 | 0.88 | 0.81 | 0.69 | 0.69 | 0.90 | 0.89 | 0.89 | 0.78 | 0.78 |
|  | ANN | 0.86 | 0.86 | 0.85 | 0.71 | 0.71 | 0.86 | 0.83 | 0.84 | 0.68 | 0.68 |
|  | SVM | 0.92 | 0.89 | 0.86 | 0.75 | 0.75 | 0.89 | 0.85 | 0.85 | 0.70 | 0.70 |
