# Supplementary material for: Climate-driven shifts in avocado suitability zones in India: Insights from ensemble modelling and niche hypervolume
Source: PLoS One. 2026 Jan 14;21(1):e0338518. doi: 10.1371/journal.pone.0338518 (PMC12803459; doi:10.1371/journal.pone.0338518)
Supplement: S4 Table — (DOCX) [file pone.0338518.s004.docx]

Supplementary Table 4. Performance of individual algorithms utilized for ESDM with NBC

|  | AUC | Sensitivity | Specificity | Kappa | TSS |
| --- | --- | --- | --- | --- | --- |
| GLM | 0.94 | 0.91 | 0.90 | 0.34 | 0.81 |
| GAM | 0.92 | 0.88 | 0.88 | 0.29 | 0.75 |
| MARS | 0.88 | 0.86 | 0.93 | 0.39 | 0.79 |
| CTA | 0.88 | 0.91 | 0.86 | 0.77 | 0.77 |
| RF | 0.92 | 0.91 | 0.88 | 0.79 | 0.79 |
| ANN | 0.92 | 0.92 | 0.87 | 0.79 | 0.79 |
| SVM | 0.94 | 0.91 | 0.94 | 0.85 | 0.86 |
