# Supplementary figures and images for: Climate-driven shifts in avocado suitability zones in India: Insights from ensemble modelling and niche hypervolume
Source: PLoS One. 2026 Jan 14;21(1):e0338518. doi: 10.1371/journal.pone.0338518 (PMC12803459; doi:10.1371/journal.pone.0338518)

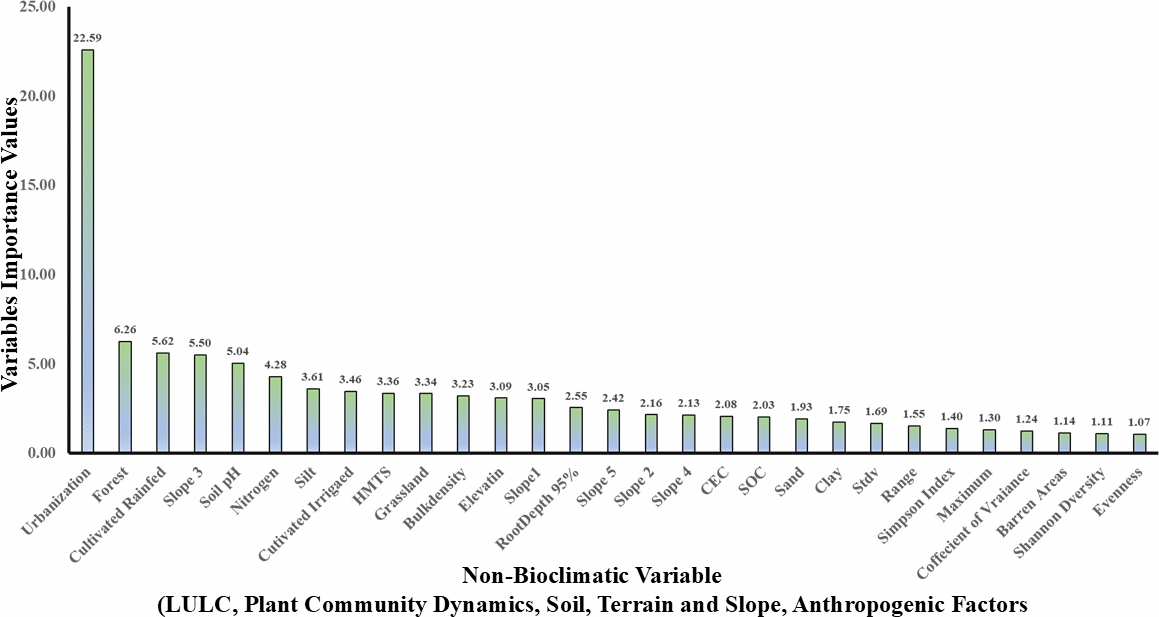

Supplement: S1 Fig — Uncertainty maps with different bioclimatic and non-bioclimatic predictors. Maps created using ArcGIS software. Source: Esri. (2024). ArcGIS Desktop: Release 10.8. Redlands, California, United States: Environmental Systems Research Institute. (TIF) [file pone.0338518.s005.tif]

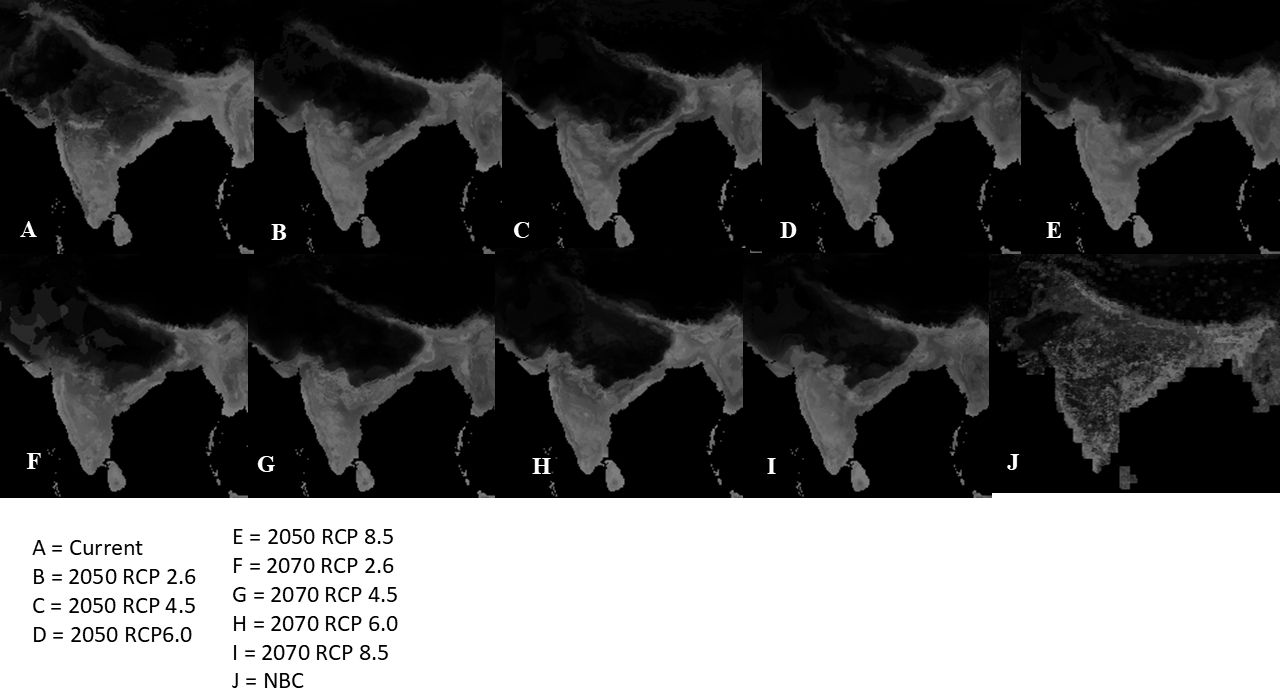

Supplement: S2 Fig — (TIF) [file pone.0338518.s006.tif]
